# A follow-up study: 6-year cART-free virologic control of rhesus macaques after PD-1-based DNA vaccination against pathogenic SHIV_SF162P3CN_ challenge

**DOI:** 10.1128/spectrum.03350-23

**Published:** 2023-11-03

**Authors:** Xiaoen He, Yik Chun Wong, Menglong Zhong, Yufei Mo, Bo Li, Lok Yan Yim, Xin Li, Wan Liu, Yanhua Du, Hui Wang, Haoji Zhang, Zhiwei Chen

**Affiliations:** 1 Department of Clinical Microbiology and Infection Control, The University of Hong Kong–Shenzhen Hospital, Shenzhen, Guangdong, China; 2 Immuno Cure Holding (HK) Limited, Hong Kong, China; 3 AIDS Institute and Department of Microbiology, School of Clinical Medicine, Li Ka Shing Faculty of Medicine, The University of Hong Kong, Hong Kong, China; 4 Department of Veterinary Medicine, Foshan University, Foshan, China; 5 HKU-AIDS Institute Shenzhen Research Laboratory and AIDS Clinical Research Laboratory, Guangdong Key Laboratory of Emerging Infectious Diseases, Shenzhen Key Laboratory of Infection and Immunity, Shenzhen Third People’s Hospital, Shenzhen, China; 6 State Key Laboratory of Emerging Infectious Diseases, The University of Hong Kong, Hong Kong, China; 7 Center for Virology, Vaccinology and Therapeutics, Hong Kong Science and Technology Park, Hong Kong, China; Joint Research Center for Human Retrovirus Infection, Kumamoto, Japan

**Keywords:** long-term therapeutic efficacy, SHIV, HIV-1/AIDS, therapeutic DNA vaccine, PD-1

## Abstract

An effective vaccine for combination antiretroviral therapy (cART)-free virologic control of HIV-1 by enhancing antigen-specific effector-memory CD8^+^ T lymphocytes could be useful in controlling the AIDS pandemic. Previously, we demonstrated that a programmed death-1 (PD-1)-based DNA vaccine, pRhPD1-p27, was effective in inducing Gag-p27-specific effector-memory CD8^+^ T cells for viremia suppression in rhesus macaques infected with pathogenic SHIV_SF162P3CN_ for 2 years. In this follow-up study, we report on two pRhPD1-p27-vaccinated and SHIV_SF162P3CN_-infected macaques that have remained alive to date with undetectable viremia levels and low proviral loads over 6 years in the absence of cART, achieving a state of sustained virologic control. Polyfunctional effector-memory Gag-p27-specific T cells were maintained in these macaques. Moreover, several T cell epitopes found at 6 years post-viral challenge were identical to those induced during the vaccination phase, indicating sustained vaccine-induced memory T cell responses. The viral challenge resulted in *de novo* Nef-specific T cell responses, which were also maintained. These Gag-p27- and Nef-specific T cell responses were stronger than those in some SHIV_SF162P3CN_-infected macaques that showed viremia control after experimental therapy using a tandem bispecific neutralizing antibody. Our findings indicate that the PD-1-based DNA vaccine strategy holds promise as a clinical immunotherapy for long-term HIV-1 suppression.

**IMPORTANCE:**

Efficient strategies for HIV-1 cART-free virologic control are critical for ending the AIDS pandemic. The essential role of effector-memory CD8^+^ T cells in controlling viremia and eliminating virus-infected cells has made them a promising target for vaccine development. It has been previously reported that PD-1-based DNA vaccination was effective in inducing polyfunctional effector-memory CD8^+^ T cells for AIDS virus control for 2 years in rhesus monkeys. This follow-up study extends the findings and shows that a viremia-free period of over 6 years was detected in two monkeys immunized with PD-1-based DNA vaccine against pathogenic SHIV_SF162P3CN_ infection in the absence of antiretroviral therapy. Long-term vaccine-induced memory T cell responses were detected. Our results warrant the clinical trials of PD-1-based DNA vaccines for achieving HIV-1 cART-free virologic control used either alone or in combination with other biomedical interventions.

## INTRODUCTION

To date, the combination antiretroviral therapy (cART) for both pre-exposure prophylaxis (PrEP) and post-exposure prophylaxis (PEP) contributes efficiently to the reduction of AIDS deaths and HIV-1 transmission cases (1, 2). However, due to the persistence of HIV-1 latent reservoirs in spite of effective cART, a complete or substained cART-free virologic control for HIV-1 infection has been a great challenge for over 40 years since the discovery of AIDS and its causative agent HIV-1 in the 1980s (3). Moreover, the extensive HIV-1 genetic diversity facilitates the formation of mutational escape variants that resist cART drugs, causing clinical failure of viral control (4). A huge number of viral variants also hamper AIDS vaccine development. Identifying novel vaccination strategies, including the induction of broadly neutralizing antibodies (bnAbs) and robust cross-reactive T cell responses will enhance the arsenal against the HIV/AIDS pandemic.

Dozens of HIV-1 vaccine candidates are currently under preclinical testing and clinical trials (1). Some of the clinical trials had to be discontinued due to their poor preventive efficacies in humans, including HVTN702/Uhambo (5, 6), HVTN705/Imbokodo (7), and HVTN706/Mosaico (8). Robust CD4^+^ T cell responses, rather than CD8^+^ T cell responses, were highlighted in HVTN702 (9). To priming virus-specific CD8^+^ T cell responses for protection, cytomegalovirus (CMV) was a promising vaccine vector to deliver antigens for sustained simian immunodeficiency virus (SIV) control in monkeys (10, 11). The safety of a potential CMV-vectored vaccine, as well as the immunogenicity and efficacy, remains to be answered in human trials (12). In addition to prophylactic use, HIV-1 vaccines may also be used in therapeutic immunizations to achieve cART-free virologic control, a stage defined by sustained viremia suppression without life-long cART in infected patients or animals.

Our team has developed a vaccine technology known as programmed death-1 (PD-1)-based DNA vaccine strategy, in which recombinant antigens expressing target antigens of interest fused to a soluble PD-1 domain is encoded by a DNA plasmid vector. Murine studies have demonstrated the dendritic cell targeting ability of the recombinant PD-1-fused antigens and the superiority of immunogenicity and protective efficacy of PD-1-based DNA vaccines against viral infection and tumorigenesis over conventional vaccine designs (13, 14). Notably, strong polyfunctional effector-memory CD8^+^ T cell responses were elicited by PD-1-based DNA vaccines (13, 15).

Recently, we reported a macaque study evaluating the potential of a PD-1-based DNA vaccine against simian–human immunodeficiency virus (SHIV) (15). Vaccination of rhesus macaques with a DNA vaccine encoding rhesus soluble PD-1-fused SIV Gag-p27 antigen, namely, pRhPD1-p27, conferred sustained viremia control against pathogenic SHIV_SF162P3CN_ challenge, mediated by strong polyfunctional vaccine-induced effector-memory CD8^+^ T responses (15). In a group of four pRhPD1-p27-vaccinated macaques, an aviremic state was maintained for 2 years, indicating that a potential cART-free virologic control could be achievable with the PD-1-based DNA vaccination. Understanding the extent of such viral suppression is valuable for HIV-1 cART-free virologic control using this unique vaccine technology.

In this study, we report a follow-up investigation on the viremia level and T cell immune responses in the previously reported pRhPD1-p27-vaccinated/SHIV_SF162P3CN_-infected macaques 6 years post-pathogenic SHIV_SF162P3CN_ challenge.

## RESULTS

### A prolonged period with undetectable viremia in the absence of cART was maintained in two of four pRhPD1-p27-vaccinated/SHIV_SF162P3CN_-infected macaques

**Fig 1:**
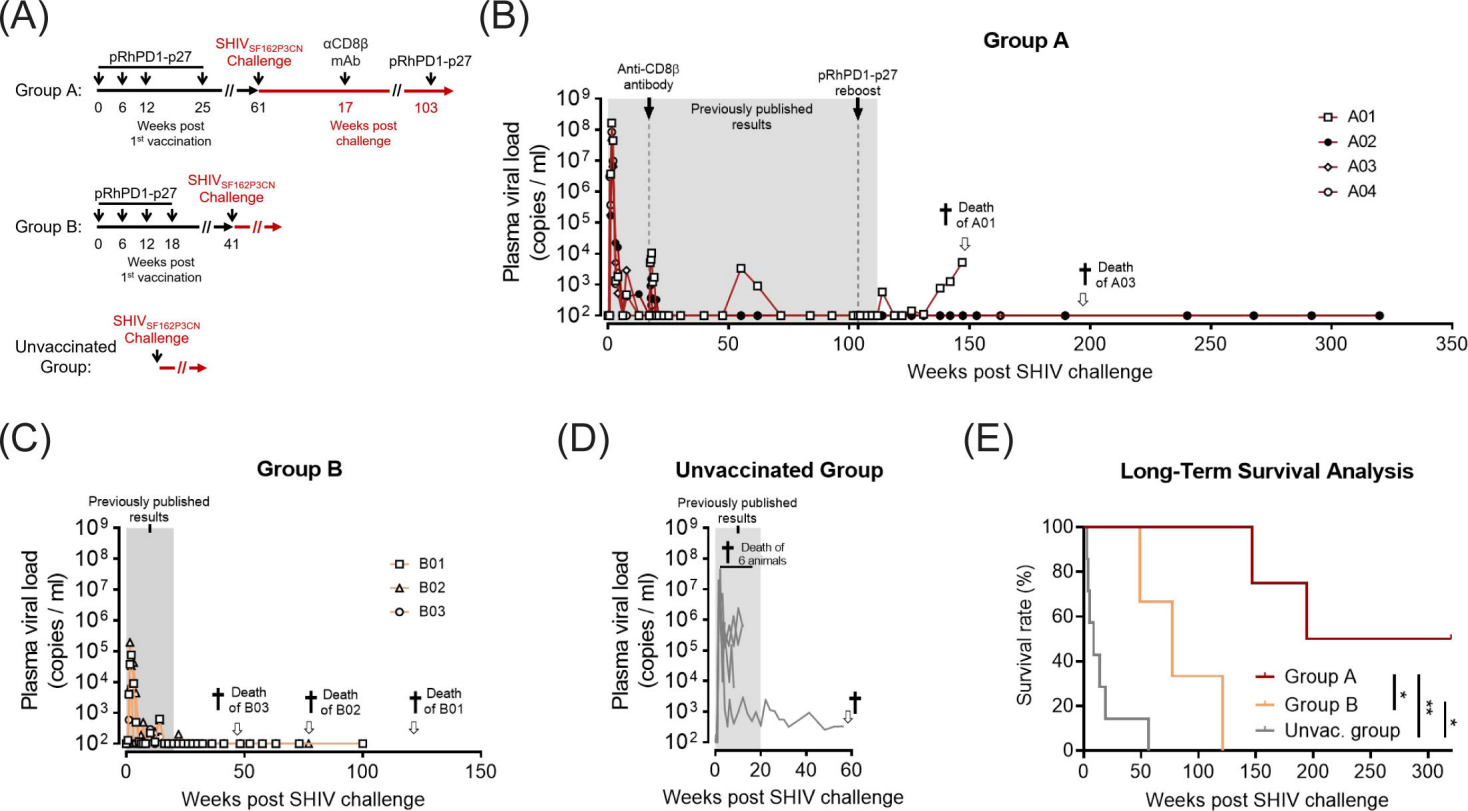
Long-term plasma viremia suppression in pRhPD1-p27-vaccinated/SHIV-challenged macaques. (**A**) Experimental design, as reported in Wong et al. (15). In Group A, four macaques were vaccinated with the PD-1-based pRhPD1-p27 DNA vaccine three times at 6-week intervals and a booster homologous vaccination at 25 weeks. In Group B, three macaques were vaccinated with the pRhPD1-p27 vaccine four times at 6-week intervals. In the unvaccinated group, unvaccinated macaques were included as controls. Macaques were challenged with SHIV_SF162P3CN_ at the times indicated. Plasma viral loads were measured over time. In Group A, macaques were treated with anti-CD8β antibody CD8b255R1 at 17 weeks post-challenge and revaccinated with pRhPD1-p27 at 103 weeks post-challenge. (**B–D**) Plasma viral loads from the macaques in Group A (**B**), Group B (**C**), and unvaccinated group (**D**) after viral challenge, with measurements up to 320 weeks post-infection. (**E**) Long-term survival analysis of the pRhPD1-p27-vaccinated (Groups A and B) and the unvaccinated macaques after SHIV_SF162P3CN_ challenge. The significance of the difference was determined by the log-rank test.

In the original publication on the PD-1-based pRhPD1-p27 DNA vaccine, three groups of Chinese-origin rhesus macaques were examined (15) (Fig. 1A): four macaques from Group A (A01–A04) received pRhPD1-p27 vaccination four times at 6–13-week intervals intramuscularly with electroporation (i.m./EP), three macaques from Group B (B01–B03) were vaccinated with pRhPD1-p27 four times at 6-week intervals via i.m./EP, and nine unvaccinated macaques served as controls in the unvaccinated group. Macaques were challenged with 5,000 TCID_50_ of the CCR5-tropic, tier-2 pathogenic SHIV_SF162P3CN_ intravenously. Virological and immunological analyses were carried out over time. After challenge, macaques in Group A were also treated with anti-CD8β depleting antibody at 17 weeks post-infection (wpi) and received pRhPD1-p27 revaccination at 103 wpi. Till the end of our published observation, the four macaques in Group A maintained a sustained viremia suppression up to 2 years after viral challenge (Fig. 1B, with previously published results highlighted in gray). As reported in the original study, macaques in Group B showed undetectable viremia by the end of the 20-week observation period after viral challenge (Fig. 1C). In contrast, most of the unvaccinated macaques consistently showed >10^4^ viral RNA copies/mL of plasma viral loads during the same period (Fig. 1D), with 67% of the unvaccinated macaques needed to be euthanized because of a poor clinical situation (significant weight loss and/or persistent diarrhea).

Here, we report our continuous observation of the macaques for up to 6 years post-viral challenge. In the unvaccinated group presented here, two of the nine unvaccinated macaques originally reported (15) were used for other investigations and were removed from the follow-up observation. Results from seven unvaccinated macaques were kept for analysis, and only one animal survived past the originally reported 20-wpi period. This remaining macaque continued to show a low level of viremia over time and was not able to survive after 60 wpi (Fig. 1D). In the vaccinated Group B, all macaques remained alive for at least 49 wpi (Fig. 1C). Over time, macaques B03, B02, and B01 were euthanized at 49, 77, and 121 wpi, respectively, because of deteriorating clinical condition. Macaques B02 and B03 showed no detectable plasma viral loads at the points of euthanasia, suggesting that the worsened clinical condition in these two animals was unlikely caused by SHIV_SF162P3CN_ infection. Similarly, no viral load was detected from the plasma sample of macaque B01 collected at 100 wpi. In the vaccinated Group A, macaques A01 and A03 were euthanized at 146 and 194 wpi, respectively, due to poor health conditions. A01 had viral rebounds after CD8^+^ T cell depletion and before death, whereas A03 had no measurable viremia before death and unfortunately died of other causes (Fig. 1B). Macaques A02 and A04, however, remain alive to date, allowing us for a long-term follow-up study. We found that these two macaques maintained undetectable plasma viral load over 327 weeks after viral challenge, in the absence of any cART (Fig. 1B), together with trends of increasing CD4^+^ T cell frequency and CD4^+^/CD8^+^ ratio in between 291 and 327 wpi (Fig. 3C and D). Statistically, the pRhPD1-p27 vaccine group conferred a significant survival advantage over the unvaccinated control group (Fig. 1E). These results showed that the PD-1-based pRhPD1-p27 DNA vaccine enhanced the survival with prolonged suppression of SHIV_SF162P3CN_ replication in the absence of cART.

### Polyfunctional effector-memory antigen-specific T cells were present in the two surviving pRhPD1-p27-vaccinated/SHIV_SF162P3CN_-infected macaques at 6 years after viral challenge

**Fig 2:**
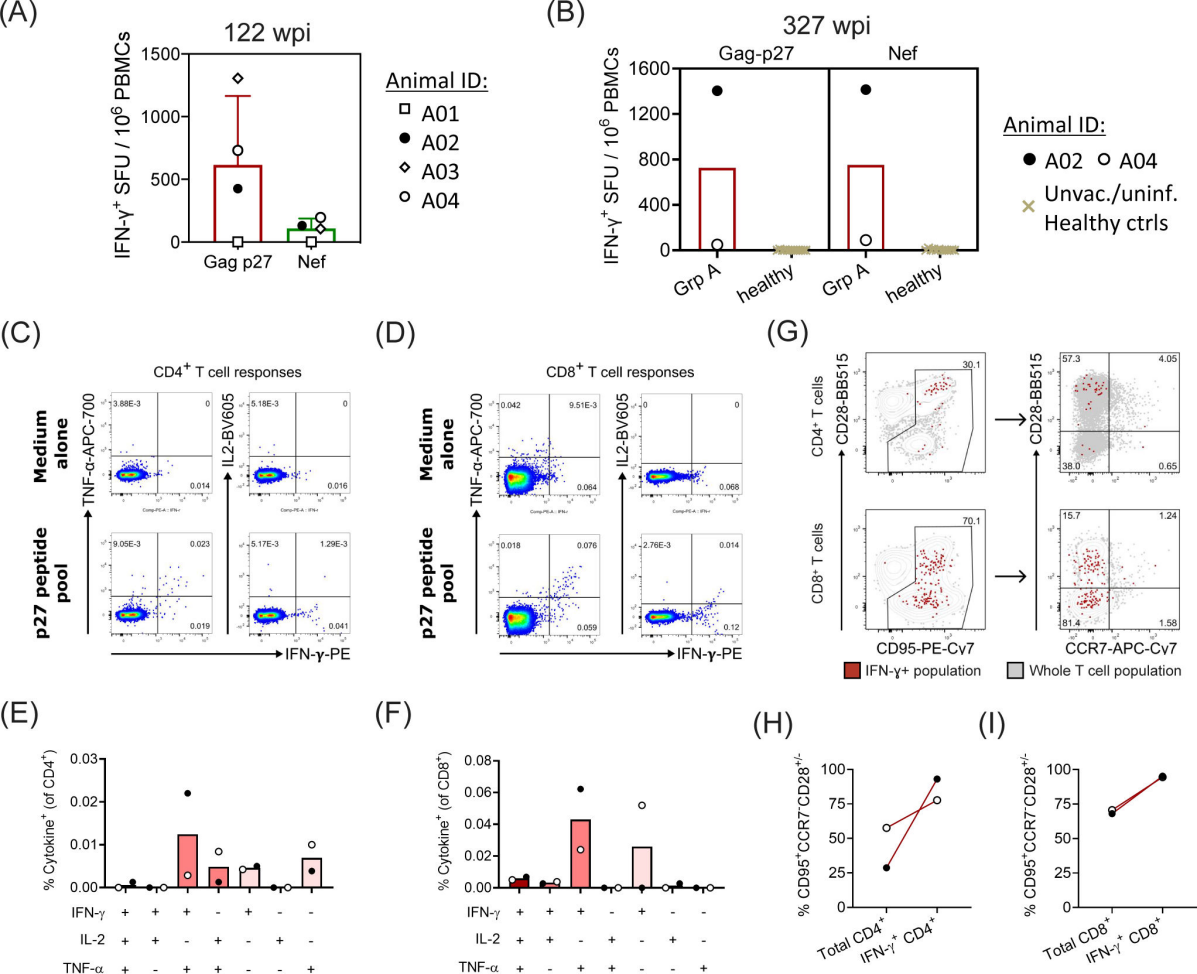
Sustained functional p27-specific effector-memory T cell responses were maintained in the pRhPD1-p27-vaccinated/SHIV-challenged macaques. PBMCs from the Group A macaques at 122 wpi (**A**) or 327 wpi (**B–I**) were stimulated with the overlapping Gag-p27 or Nef peptide pools. Antigen-specific T cell responses were then measured with IFN-γ ELISpot assays or with flow cytometric intracellular cytokine staining assays. (**A**) T cell responses as measured with IFN-γ ELISpot assays from all four Group A macaques at 122 wpi. (**B**) T cell responses as measured with the IFN-γ ELISpot assay from the macaques A02 and A04 of Group A and 12 healthy unvaccinated/uninfected macaques. (**C–D**) Representative FACS plots showing CD4^+^ T cells (**C**) and CD8^+^ T cells (**D**) that produced various cytokines after *ex vivo* stimulation with medium alone (upper) or with p27 peptide pool (lower). (**E–F**) Frequencies of CD4^+^ T cells (**E**) and CD8^+^ T cells (**F**) that produced different combinations of IFN-γ, TNF-α, and IL-2 cytokines after stimulation with p27 peptide pool. (**G**) Representative FACS plots showing the surface phenotypes of p27-specific CD4^+^ (upper) and CD8^+^ (lower) T cells. (**H–I**) Frequencies of T_EM_ (CD95^+^CCR7^−^CD28^+/−^) were compared between the overall and IFN-γ-producing cells (i.e., p27-specific T cells) within the CD4^+^ T cell (**H**) and CD8^+^ T cell (**I**) populations.

Our previous study demonstrated that CD8^+^ T cell responses induced by the pRhPD1-p27 vaccine were needed to control SHIV replication (Fig. 1A) (15). The interferon-γ (IFN-γ) ELISpot assay was first conducted to determine the Gag-p27- and Nef-specific T cells at 122 wpi for all Group A macaques (Fig. 2A). There were no viral-specific T cell responses in macaque A01 at the time point just before viral rebound in this macaque at 122 wpi. In contrast, measurable Gag-p27- and Nef-specific T cell responses were detected in the other 3 Group A macaques (Fig. 2A). Thus, viral rebound was associated with the loss of anti-viral T cell responses, and this is a potential cause of death for macaque A01. We further determined the antigen-specific T cell responses in these two long-term aviremic macaques at 327 wpi. Relatively higher frequencies of both the Gag-p27- and Nef-specific T cells were determined from A02 than A04, whereas virtually no SHIV-specific T cell responses were found in healthy unvaccinated/uninfected macaques (Fig. 2B). Notably, Nef antigen was not encoded within the pRhPD1-p27 vaccine, and thus, the induced T cell responses against Nef in macaques A02 and A04 represented *de novo* immune responses to SHIV_SF162P3CN_ infection.

To better understand the T cell immune responses in macaques A02 and A04, the polyfunctionality, including the expression of IFN-γ, tumor necrosis factor-α (TNF-α), and interleukin-2 (IL-2), of the Gag-p27-specific T cells was determined with an intracellular cytokine staining (ICS) assay as we previously described (15). Dual-functional T cells producing both IFN-γ and TNF-α formed the majority of Gag-p27-specific CD8^+^ and CD4^+^ T cells (Fig. 2C-F). Furthermore, the memory phenotypes of the Gag-p27-specific T cells were also evaluated. In line with our previous study, we found that there were still over 70% IFN-γ-producing CD8^+^ or CD4^+^ T cells having the effector-memory T cell (T_EM_) phenotype (CD95^hi^CCR7^lo^CD28^hi/lo^) after *ex vivo* Gag-p27 stimulation (Fig. 2G-I). Taken together, these results indicated that long-term, polyfunctional, effector-memory antigen-specific T cells were maintained in these two surviving macaques.

### The pRhPD1-p27-vaccinated macaques possessed advantages in viremia control and long-term vaccine-induced T cell responses

**Fig 3:**
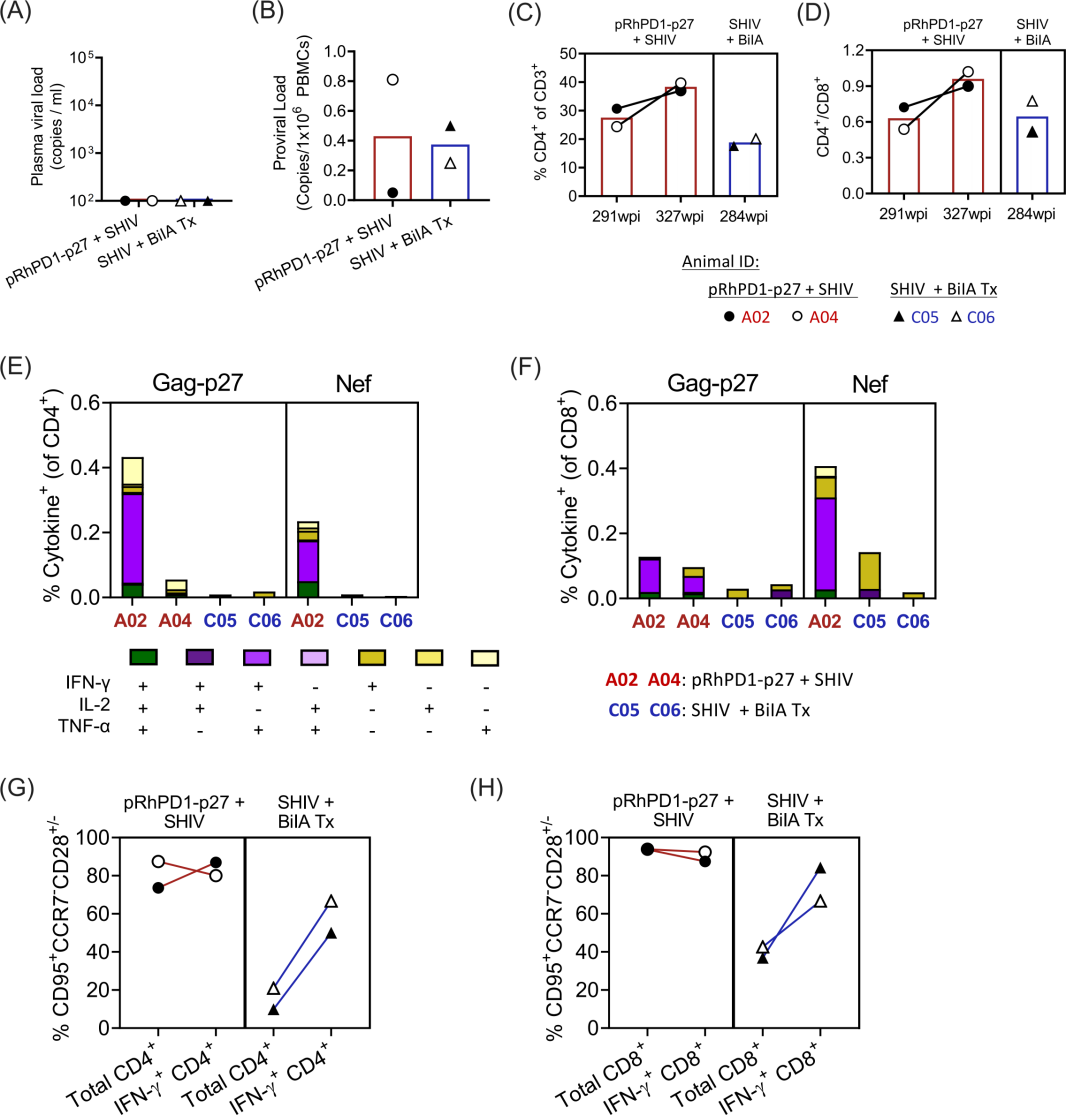
Virological and cellular immunological comparison in macaques that received pRhPD1-p27 vaccination followed by SHIV challenge and SHIV-infected macaques that received bispecific neutralizing antibody BiIA treatment at 1 day post-SHIV challenge. Comparisons of (**A**) plasma viral loads and (**B**) proviral loads in PBMCs at 319 wpi of macaques A02 and A04 that received the pRhPD1-p27 vaccine and at 269 wpi of C05 and C06, the two SHIV-infected macaques that received bispecific neutralizing antibody BiIA treatment at 1 day post-SHIV challenge [C05 and C06 were described in (16)]. (**C**) Percentages of CD4^+^ cells of CD3^+^ T cells and (**D**) ratios of CD4^+^/CD8^+^ T cells of macaques A02 and A04 at 291 and 327 wpi, as well as macaques C05 and C06 at 284 wpi. Polyfunctionality analysis of Gag-p27-specific (**E**) CD4^+^ and (**F**) CD8^+^ T cell responses induced in the two groups of macaques (pRhPD1-p27+SHIV: 291 wpi; SHIV+BiIA Tx: 284 wpi), as measured with flow cytometric intracellular cytokine staining assays. Phenotypes of Gag-p27-specific (**G**) CD4^+^ and (**H**) CD8^+^ T cells induced in the macaques indicated.

Owing to the lack of surviving macaques in the unvaccinated and infected control groups, we compared the aforementioned two macaques to two SHIV-infected controllers that received bispecific neutralizing antibody BiIA-SG treatment at day 1 post-challenge (macaques C05 and C06). The BiIA-SG-treated macaques C05 and C06 were previously reported (16). Macaques C05 and C06 survived for over 284 wpi after challenge with the same dose of 5,000 TCID_50_ of SHIV_SF162P3CN_ (Fig. S1). At the most recent sampling time points, the plasma viral load of A02, A04, C05, and C06 had undetectable plasma viral loads (Fig. 3A). In terms of proviral loads, A02 had the lowest proviral load at 0.05 copies per 1 × 10^6^ PBMCs, with the other three macaques maintaining a proviral load at the level of 0.25–0.81 copies per 1 × 10^6^ PBMCs (Fig. 3B). Trends of better CD4^+^ T cell frequency and CD4^+^/CD8^+^ T cell ratio were found in the two RhPD1-p27-vaccinated macaques than the two BiIA-treated macaques at the last measurement time point (Fig. 3C and D). Overall, pRhPD1-p27 vaccination showed a trend of similar or even better viral control, as compared to an experimental treatment using a highly effective broadly neutralizing antibody.

T cell immunogenicity against viral antigens was then compared among these monkeys. A02 and A04 showed higher frequencies of functional p27-specific effector-memory CD4^+^ and CD8^+^ T cell responses than C05 and C06 (Fig. 3E and F). Particularly, A02 had the highest percentage of polyfunctional CD4^+^ and CD8^+^ T cells specific against both Gag-p27 and Nef. A similar level of functional Nef-specific effector-memory T cell responses was found in A02 as compared to those of Gag-p27 (Fig. 3E and F). Consistently, the proportion of effector-memory Gag-p27-specific T cells (CD95^hi^CCR7^lo^CD28^hi/lo^) in A02 and A04 were higher than C05 and C06, within both the CD4^+^ and CD8^+^ T cell subsets (Fig. 3G and H). Thus, pRhPD1-p27 vaccination showed a trend of better long-term T cell responses useful for viral suppression, as compared with BiIA-SG treatment.

**Fig 4:**
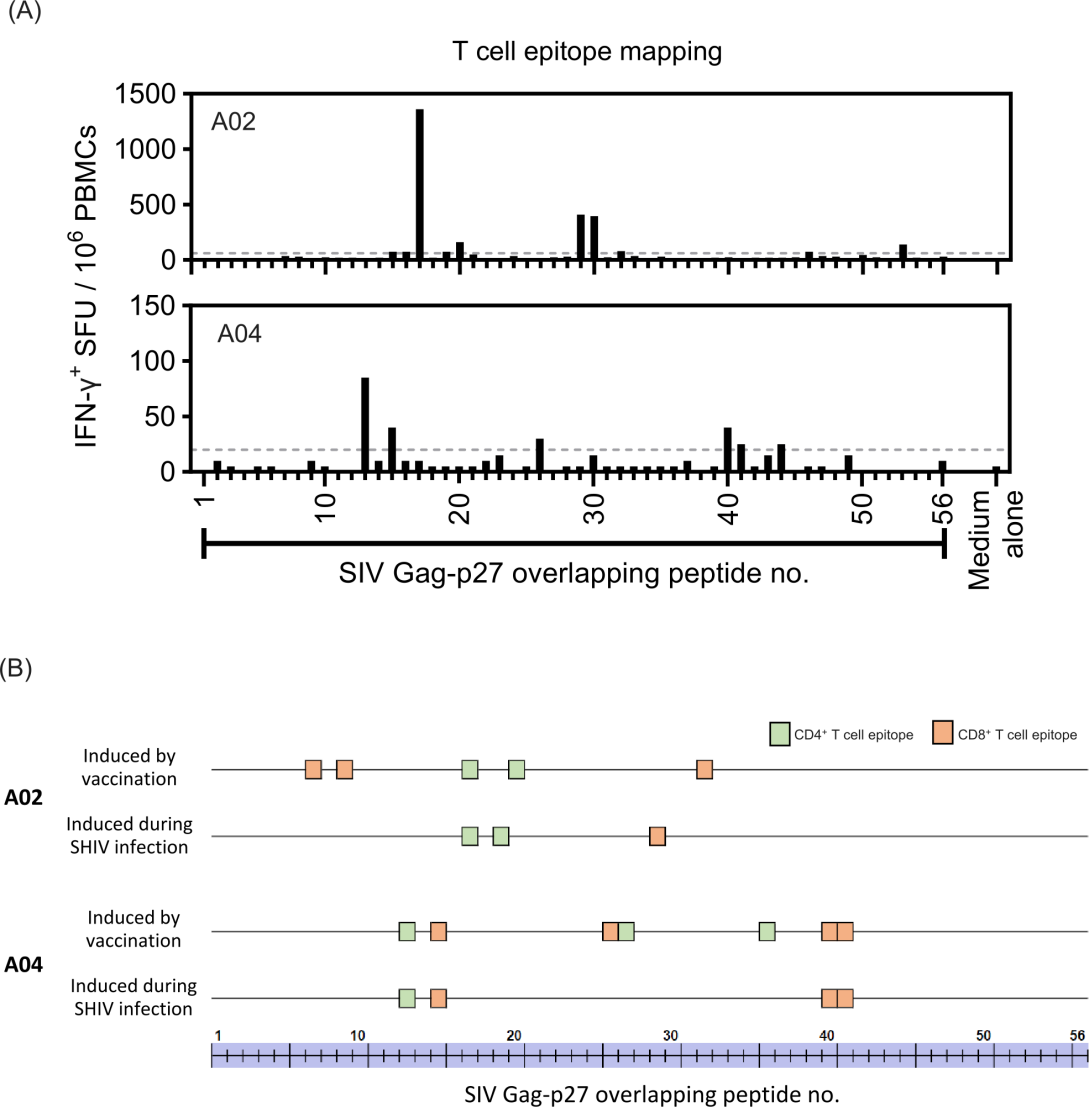
T cell epitope mapping analysis of the Gag-p27 antigen in the pRhPD1-p27 vaccinated/SHIV-infected macaques. PBMCs from the two pRhPD1-p27 vaccinated/SHIV-infected macaques, A02 (upper) and A04 (lower), respectively, were stimulated with individual overlapping 15-mer peptides spanning the Gag-p27 antigen in IFN-γ ELISpot assays. Peptides that successfully induced responses in IFN-γ ELISpot assays were then tested for their ability to induce IFN-γ production in T cells by ICS assays. (**A**) IFN-γ ELISpot data and (**B**) epitope mapping analysis for the comparison of CD4^+^ and CD8^+^ T cell epitopes identified during the vaccination phase [as reported in Wong et al. (15)] and at 327 weeks post-SHIV challenge in macaques A02 and A04. CD4^+^ and CD8^+^ T cell epitopes are represented by green and orange boxes, respectively.

The breadth of Gag-specific T cell responses is associated with better HIV-1 viremia suppression (17, 18). We previously showed that pRhPD1-p27-induced T cells targeted various regions of the viral Gag-p27 capsid antigen in vaccinated macaques (15). Here, in-depth epitope mapping analysis was conducted to examine whether T cell epitopes in A02 and A04 identified after more than 6 years post-initial vaccination could be matched with the epitopes previously identified during the vaccination stage, by using individual overlapping 15-mer peptides spanning the entire SIV Gag-p27 capsid antigen (Fig. 4A and B). Three minimal T cell epitopes were identified from A02 and A04 at 327 wpi based on the combined ELISpot and ICS analysis, and were less than those detected from these two macaques during the vaccination phase. Critically, matched T cell epitopes were found from both macaques (one matched minimal CD4^+^ T cell epitope for A02; one CD4^+^ and two CD8^+^ minimal T cell epitopes for A04). These findings demonstrated that T cell responses induced by PD-1-based DNA vaccination remained responsive after 6 years post-viral challenge.

## DISCUSSION

In this follow-up study, we demonstrated that PD-1-based pRhPD1-p27 DNA vaccine induced prolonged viral suppression in rhesus macaques, resulting in a survival period of more than 6 years after challenge with the CCR5-tropic, neutralization tier-2 pathogenic SHIV_SF162P3CN_. The viremia-free period reported here was in a similar timeframe demonstrated in the aviremic status achieved in the “Miami Monkey” that received treatment with adeno-associated viruses (AAVs) expressing three broadly neutralizing antibodies after SHIV-AD8 challenge (19, 20). AAV-vectored neutralizing antibody delivery treatment requires the persistent presence of neutralizing antibodies to sustain viral control. In contrast, the PD-1-based DNA vaccination conferred cART-free virologic control in macaques via the elicitation of strong polyfunctional effector-memory antigen-specific T cell responses in the absence of antiviral drug or neutralizing antibody therapy. Mechanistically, CD8^+^ T cell responses are critical to suppress viral replication in our model, as demonstrated by an *in vivo* CD8^+^ T cell depletion study as we previously reported (15). In macaques A02 and A04, antigen-specific T cells with an effector-memory phenotype were maintained long-term, even after 6 years post-vaccination. Moreover, several T cell epitopes identified from the vaccination phase in these two macaques remained immunogenic long after the SHIV challenge.

As compared to the two aviremic macaques, C05 and C06, that received tandem bispecific neutralizing BiIA-SG antibody treatment (16), there was a trend of higher magnitude of anti-viral T cell immunity in macaques A02 and A04. In addition, more than 70% of responding CD8^+^ T cells after Gag-p27 stimulation from macaques A02 and A04 were polyfunctional and produced at least two cytokines while 0% and 60% of responding CD8^+^ T cells from macaques C05 and C06 were able to express at least two cytokines, respectively. It indicated a trend of better T cell functionality in macaques A02 and A04. The beneficial effect of PD-1-based DNA vaccination against SHIV challenge was also reflected by the *de novo* generation and prolonged maintenance of anti-Nef T cell responses after SHIV infection, demonstrating the preservation of reactive T cells in the vaccinated macaques after viral challenge.

Although similarly aviremic, the anti-viral T cell responses in macaques A02 and A04 were different from each other. For example, the anti-p27 and anti-Nef T cell responses induced in A02 were both higher than those in A04. One possibility is that the higher maintenance level of antigen-specific T cell responses in A02 was induced by the spontaneous expression of viral antigens from latently infected cells (21). However, the proviral load in PBMCs of A02 was the lowest among all five macaques examined, suggesting that the proviral level might unlikely be required to sustain high virus-specific T cell responses in this animal. Although the small sample size limited the determination of the association between proviral loads and the magnitudes of T cell responses, it is possible that sustained high T cell responses in A02 might facilitate the elimination of latently infected cells and limit the size of the viral reservoir.

Recently, the late-stage clinical trials focusing on a prophylactic HIV vaccine approach using an adenovirus vector to deliver mosaic viral antigen designed *in silico* have been discontinued due to the lack of preventive efficacy against HIV infection (7, 8). The induction of broadly neutralizing antibody responses against diverse HIV-1 subtypes has been an ongoing research focus for designing new preventive HIV vaccine candidates (1, 22, 23). In comparison, the generation of long-lasting T cell responses aiming to facilitate the elimination of viral-infected, antigen-producing cells has been evaluated as a main strategy for therapeutic HIV vaccines (24 – 26). A recent phase 1 trial on the HIVACAT T cell immunogen (HTI) vaccine regimen indicated a significant association of antigen-specific T cell responses inducing the vaccination with extended time period without cART treatment during antiretroviral treatment interruption (ATI) and lower viremia level at the end of ATI (25), supporting the clinical development of PD1-based therapeutic T cell vaccines for HIV-1 cART-free virologic control.

One limitation of this study is the small number of macaques that received the PD-1-enhanced DNA vaccine. However, the long-term follow-up of macaques demonstrates that the use of a PD-1-based DNA vaccine has the potential to induce viral-specific T cell responses that achieve cART-free virologic control for the AIDS virus. Of the four pRhPD1-p27-vaccinated/SHIV-challenged macaques in Group A, two monkeys, macaques A02 and A04, continue to survive with undetectable viral loads. In comparison, macaques A01 and A03 needed to be euthanized. Macaque A01 survived up to 146 weeks post-viral challenge. Its plasma viral load gradually increased starting from 125 weeks post-viral challenge and reached approximately 5 × 10^3^ viral RNA copies/mL in plasma at the point of euthanasia. The second macaque A03 was euthanized at 194 weeks post-challenge due to a sudden poor health situation. Since this event occurred during the lockdown period of the COVID-19 pandemic, neither viral load examination nor autopsy could be conducted. At 189 weeks post-infection, just 5 weeks before euthanasia, A03 remained aviremic, and thus, the cause of the death of A03 was possibly unrelated to SHIV infection. A difference in the survival rate was observed between Group A and Group B. A difference in the pRhPD1-p27 vaccination regimen between the two vaccination groups might influence long-term clinical outcomes. In addition, animals in Group A had been treated with anti-CD8β antibody to deplete CD8^+^ T cells and were revaccinated with pRhPD1-p27 at around 2 years post-challenge. The CD8 depletion procedure affected the short-term virological and immunological profiles as previously reported (15) but should not provide any positive influence on the clearance of viremia seen in Group A. In contrast, the booster vaccination at 2 years post-challenge augmented the anti-Gag-p27 memory CD8^+^ T cell responses. This booster vaccination procedure aimed to evaluate the outcome of vaccination during the chronic stage and in principle might potentially extend the aviremic condition in the macaques, as intended. This finding would guide the design of the dosing strategy for therapeutic vaccination.

It should be noted that A02 and A04 possessed a potential protective MHC-I allele Mamu-B*017 (15). Mamu-B*017^+^ macaques had reduced chronic viral loads by 10–20-fold irrespective of the vaccination status (27 – 29). It should be noted that most of the Mamu-B*17^+^ macaques remained viremic chronically, with measurable >200 viral RNA copies/mL in plasma during chronic SIV infection. Approximately only 10% of macaques may show viral load <200 copies/mL in plasma during the chronic infection phase (29). Another study evaluated vaccination outcomes in Mamu-B*017^+^ macaques and found that only one of the eight unvaccinated Mamu-B*017^+^ macaques showed spontaneously controlled SIV infection (30). The vaccination of Mamu-B*017^+^ macaques with vif, rev, tat, and nef antigens using various vectors induced antigen-specific CD8^+^ T cells, which did not facilitate long-term virologic suppression (30). This conclusion is different from our studies, which demonstrated the role of vaccine-elicited CD8^+^ T cells by PD-1-based DNA vaccine in controlling viral replication. In addition, no antigenic epitopes that can be recognized by Mamu-B*017-restricted T cells have been identified on the SIV Gag antigen (31). As our PD-1-based DNA vaccine used Gag-p27 as the antigen, it is unlikely that the expression of Mamu-B*017 dictates the prolonged viral suppression and extended survival in our model. Notably, like macaque A03, three macaques in group B (B01-B03) were not died of viral rebound, strengthening the efficacy of PD-1 based DNA vaccinations for prolonged cART-free virologic control.

To support human clinical use, a PD-1-based DNA vaccine encoding a PD-1-linked bivalent mosaic HIV-1 Gag antigen, namely, ICVAX (32), has been developed. By combining the PD-1-based DNA vaccine technology and the mosaic antigen design strategy used in this vaccine, it has been demonstrated that strong, broadly reactive, and poly-functional antigen-Gag T cell responses can be elicited in preclinical macaque studies. The ICVAX vaccine is currently in a phase 1 clinical trial to evaluate the safety and the immunogenicity of this novel therapeutic vaccine platform in HIV patients (China Clinical Trial registration CTR20223007).

In conclusion, this study contributes further evidence that PD-1-based DNA vaccination represents a promising strategy for developing a potential therapeutic AIDS vaccine through the generation of long-lasting viral-specific T cell responses to achieve cART-free virologic control status.

## MATERIALS AND METHODS

### Animals and study design

Outbred Chinese-origin, female rhesus macaques were housed at the Foshan University Animal Research Center and were free of simian immunodeficiency virus infection before experiments.

Macaques A01–A04 and B01-B03, which were vaccinated with the PD-1-based pRhPD1-p27 DNA vaccine followed by challenging with SHIV_SF162P3CN_, were reported in Wong et al. (15). The seven control macaques, which were challenged with SHIV_SF162P3CN_, were also reported in (15). Macaques C05 and C06, which were challenged with SHIV_SF162P3CN_ followed by treatment with tandem bispecific neutralizing BiIA antibody at day 1 post-challenge, were described in (16).

### Viral quantitation assays

Plasma viral loads were determined as previously described (15). In brief, viral RNA isolated from plasma using a QIAamp Viral RNA Minikit (Qiagen) was reverse-transcribed into cDNA using a PrimeScript RT Reagent kit (Takara) with random hexamers. The plasma viral RNA copy number was then determined with a SYBR green-based real-time quantitative PCR (Takara) using SIV-Gag-specific primers 5′-GTAGTATGGGCAGCAAATGAAT-3′ and 5′-CACCAGATGACGCAGACAGTAT-3′. To determine cell-associated DNA viral load, DNA was first isolated from PBMC using a QIAamp DNA Mini Kit (Qiagen). Real-time quantitative PCR was then performed with the DNA samples to determine the proviral DNA copies using the SIV-Gag-specific primers listed above, with the results normalized based on albumin gene copy numbers determined from the DNA samples.

### Cellular immune assays

PBMCs from rhesus macaques were isolated using Ficoll density gradient centrifugation and were used in IFN-γ ELISpot or intracellular cytokine staining assays to examine T cell immune responses, as previously described (15, 32). In ELISpot assays, 2 × 10^5^ PBMCs were stimulated with 1 µg/mL of 15-mer overlapping peptide pools spanning the SIVmac239 Gag-p27 and Nef antigens (NIH AIDS Reagents Program), and the T cell responses were determined using Macaque IFN-γ T cell ELISpot kit (Mabtech). Backgrounds were determined from PBMCs without peptide stimulation. In ICS assays, up to 1 × 10^6^ PBMCs were stimulated with 1 µg/mL of 15-mer overlapping Gag-p27 or Nef peptide pools in the presence of 0.5 µg/mL of anti-CD28 and anti-CD49d antibodies (BioLegend). After a 2-hour incubation at 37°C with 5% CO_2_, brefeldin A (7.5 µg/mL; Sigma-Aldrich) was added, and the cells were further incubated at 37°C with 5% CO_2_ overnight. Cells were then surface-stained, fixed, and permeabilized using the Cytofix/Cytoperm kit (BD Biosciences) and stained for intracellular cytokines. The following antibodies were used: anti-CD3 (SP34-2; Horizon V450), anti-CD4 (OKT4; PerCP-Cy5.5), anti-CD8α (RPA-T8; APC), anti-CD28 (CD28.2; FITC), anti-CD95 (DX2; PE-Cy7), anti-CCR7 (G043H7; APC-Cy7), anti-IFN-γ (B27; BV605, PE), anti-TNFα (Mab11; AF700), and anti-IL-2 (MQ1-17H12; BV605). The antibodies were from BD Biosciences or BioLegend. Zombie Aqua fixable viability stain was used during the surface staining step to discriminate against dead cells. Data were acquired with a BD FACSymphony A3 instrument and analyzed using FlowJo v10.7 software. Backgrounds were determined from co-stimulated cells without peptide stimulation. The presented IFN-γ ELISpot and ICS results were subtracted with the background values, unless indicated otherwise. In epitope mapping analysis, responses toward individual 15-mer peptides (1 µg/mL) were determined using IFN-γ ELISpot assays, with peptides that provided positive signals (4× background levels) were further examined with ICS assays. Percentages of CD4^+^ T cells of total CD3^+^ T cells and CD4^+^/CD8^+^ T cell ratios were determined also by flow cytometric analysis.

### Statistical analysis

Data were analyzed using Prism v8 (GraphPad). Survival analysis was performed using the log-rank test. The probability value (*P*) of less than 0.05 was considered statistically significant.

## SUPPLEMENTAL MATERIAL

Fig. S1 spectrum.03350-23-s0001.pdf Long-term plasma viral load analysis of the two SHIV-infected monkeys treated with bispecific neutralizing BiIA antibody at day 1 post-infection (C05 and C06).

ASM does not own the copyrights to Supplemental Material that may be linked to, or accessed through, an article. The authors have granted ASM a non-exclusive, world-wide license to publish the Supplemental Material files. Please contact the corresponding author directly for reuse.
