# Supplementary material for: A follow-up study: 6-year cART-free virologic control of rhesus macaques after PD-1-based DNA vaccination against pathogenic SHIVSF162P3CN challenge
Source: Microbiol Spectr. 2023 Nov 3;11(6):e03350-23. doi: 10.1128/spectrum.03350-23 (PMC10715146; doi:10.1128/spectrum.03350-23)
Supplement: Fig. S1 — Long-term plasma viral load analysis of the two SHIV-infected monkeys treated with bispecific neutralizing BiIA antibody at day 1 post-infection (C05 and C06). [file spectrum.03350-23-s0001.pdf]

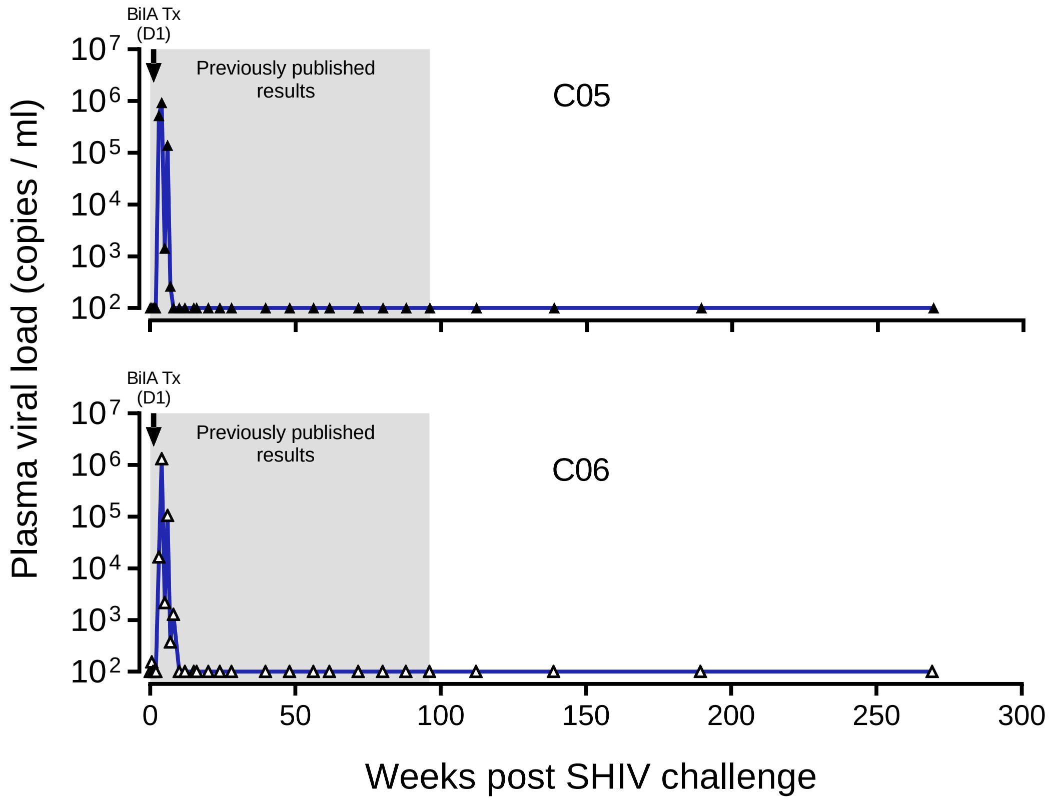

Supplementary Figure 1: Long term plasma viral load analysis of the two SHIV-infected monkeys treated with bi-specific neutralizing BiIA antibody at day 1 post infection (C05 and C06).
